# LINC01197 inhibits influenza A virus replication by serving as a PABPC1 decoy

**DOI:** 10.1186/s13567-024-01379-7

**Published:** 2024-09-27

**Authors:** Yihe Wang, Ning Shi, Hansi Zhang, Jinna Luo, Hongjian Yan, Huiyan Hou, Zhenhong Guan, Lili Zhao, Ming Duan

**Affiliations:** 1 State Key Laboratory for Diagnosis and Treatment of Severe Zoonotic Infectious Diseases, Key Laboratory for Zoonosis Research of the Ministry of Education, Institute of Zoonosis, and College of Veterinary Medicine, Jilin University, 130062 Changchun, Jilin Province China; 2 College of Basic Medical Sciences, Jilin University, 130021 Changchun, Jilin Province China

**Keywords:** Influenza A virus, LncRNA, LINC01197, replication

## Abstract

Influenza A viruses (IAVs) significantly impact animal and human health due to their zoonotic potential. A growing body of evidence indicates that the host’s long noncoding RNAs (lncRNAs) play crucial roles in regulating host–virus interactions during IAV infection. However, numerous lncRNAs associated with IAV infection have not been well characterised. Here, in this study, we identify the LINC01197 as an antiviral host factor. LINC01197 was significantly upregulated after IAV infection, which is controlled by the NF-κB pathway. Functional analysis revealed that overexpression of LINC01197 inhibited IAV replication and virus production, while knockdown of LINC01197 facilitated IAV replication. Mechanistically, LINC01197 directly interacts with poly(A) binding protein cytoplasmic 1 (PABPC1), which in turn sequesters and restricts its functions. This work shows that LINC01197 acts as a protein decoy, suppressing IAV replication and playing a key role in controlling IAV replication.

**Supplementary Information:**

The online version contains supplementary material available at 10.1186/s13567-024-01379-7.

## Introduction

Influenza A virus (IAV) is an enveloped, negative-sense single-stranded, segmented RNA virus of the family *Orthomyxoviridae* that circulates naturally in animals, including birds, pigs, bats, dogs and cats, marine mammals, and humans [1–3]. IAV, a zoonotic pathogen, enables interspecies transmission, reassortment, and the emergence of novel pandemics. A novel swine-origin IAV (H1N1) emerged in 2009, demonstrating this [4]. While these viruses affect animal health, including livestock and domestic poultry, they also pose an ongoing threat to global human health, with the potential to cause severe disease and life-threatening complications in high-risk groups.

Although studies on virus-host interactions have mainly focused on protein factors, non-coding RNAs (ncRNAs) have emerged as critical factors involved in viral replication, pathogenesis and host antiviral immune responses. Examples include microRNAs (miRNAs) and long non-coding RNAs (lncRNAs). IncRNAs are a class of RNA molecules with more than 200 nucleotides, and our understanding of their importance and function has significantly increased in recent years [5]. Based on their genomic location, most lncRNAs can be categorised into intergenic lncRNAs (lincRNAs), anti-sense lncRNAs, sense lncRNAs, intronic lncRNAs, bi-directional lncRNAs and enhancer lncRNAs [6]. It is well known that although lncRNAs have limited coding potential, they can function as signals, decoys, guides, or scaffolds through their primary sequences and higher-order structures [7]. Through direct interaction with DNA, RNA, or proteins, lncRNAs have the potential to mediate various biological processes, including epigenetics, chromatin remodelling, transcription, apoptosis, response to viruses, and immune response [8].

Evidence increasingly suggests that host lncRNAs help regulate IAV replication or the host antiviral pathway. Several lncRNAs have been reported to directly regulate IAV replication by acting on viral components. LncRNA PAAN promotes the assembly of functional viral RNA-dependent RNA polymerase (RdRp, consisting of PB1, PB2, and PA), thereby enhancing viral RNA synthesis [9]. By directly interacting with viral PB1, IPAN, an interferon (IFN)-independent lncRNA, stabilises the PB1 protein, which in turn enhances the efficiency of viral RNA synthesis [10]. Lnc45, as a broad-spectrum antiviral factor, possibly hampers IAV replication by inhibiting polymerase activity and preventing the nuclear accumulation of NP and PA via its stem ring arms [11]. However, most lncRNAs modulate IAV infection by regulating intracellular signalling and gene expression. For example, lncRNA-155, which is derived from MIR155HG, promotes innate immune responses to IAV infection by regulating the expression of protein tyrosine phosphatase 1B (PTP1B) in infected cells [12]. Acting as a molecular scaffold, lnczc3h7a facilitates tripartite motif-containing protein 25 (TRIM25)-mediated K63-linked ubiquitination of RIG-I, which supports a more potent innate immune response against RNA viruses, including IAV [13]. Lnc-Cxcl2 attenuates IAV-induced lung inflammation by binding to the *Cxcl2* promoter and maintaining its repressed chromatin state *in cis* [14]. Direct interaction with glutamate oxaloacetate transaminase (GOT2) by lncRNA ACOD1 effectively enhances GOT2 catalytic activity and the production of its metabolites, thereby promoting IAV replication [15]. Notably, although IAV induces numerous differentially expressed lncRNAs in host cells, only a limited number of studies have validated their roles in the IAV-host interplay. Hence, the specific roles of numerous host lncRNAs in the progression of IAV infection remain to be clarified.

A previous study has shown that LINC01197 (ENST00000508732) is significantly induced by hepatitis C virus (HCV) infection [16]. However, the expression of LINC01197 in response to different viruses, its effect on viral replication, and the detailed molecular mechanisms involved are yet to be established. This study demonstrates that LINC01197 is an IAV-induced lncRNA controlled by the NF-κB pathway in A549 cells. LINC01197 exerts a significant inhibitory effect on IAV replication. Further mechanistic studies reveal that LINC01197 interacts with poly(A) binding protein cytoplasmic 1 (PABPC1) and competes with viral mRNA for PABPC1 binding, reducing IAV replication. These findings indicate that LINC01197 functions as an inducible antiviral host factor by acting as a protein decoy for PABPC1.

## Materials and methods

### Cells, cell culture, and cell fractionation

The following cells were grown in Dulbecco’s Modified Eagle’s Medium (DMEM) or RPMI1640 (Corning, Tewksbury, MA, USA), supplemented with 10% fetal bovine serum (FBS; HyClone, Logan, UT, USA) and antibiotics (penicillin and streptomycin) (Invitrogen, Carlsbad, CA, USA) at 37 °C under 5% CO_2_ atmosphere: Human lung epithelial cells (A549), human cervical cancer cells (Hela), human acute monocytic leukaemia cells (THP-1), human neuroblastoma cells (SH-SY5Y), human non-small cell lung cancer cells (H1299), and Madin-Darby canine kidney cells (MDCK). Following the manufacturer’s protocol, the cytoplasmic and nuclear fractions were separated using a PARIS kit (Invitrogen).

### Viruses, viral infection, and virus titres assay

Influenza virus strains, including A/Puerto Rico/8/34 (PR8), A/WSN/33 (WSN), and A/Lufang/9/93 (H3N2), were cultured in specific pathogen-free (SPF) embryonated chicken eggs. Unless otherwise indicated, cell monolayers were washed and incubated with IAV at a multiplicity of infection (MOI) of one for 1 h in a medium containing 2 µg/mL *N*-tosyl-l-phenylalanine chloromethyl ketone (TPCK)-treated trypsin (Sigma-Aldrich, St. Louis, MO, USA), and then cultured at 37 °C in fresh DMEM. The cell culture supernatant was collected at the indicated intervals following viral infection. After diluting the supernatant several times, virus titres were determined on MDCK cells using a 50% tissue-culture infectious dose (TCID_50_).

### Regents and cell stimulation

Polyinosinic-polycytidylic acid (polyI: C)-LMW was obtained from InvivoGen (San Diego, CA, USA). Human IFN-β (300-02BC) and IFN-α2A (300-02AA) recombinant proteins were purchased from PeproTech (Cranbury, NJ, USA). BAY11-7082 (MedChemExpress, Monmouth Junction, NJ, USA), a potent and selective NF-κB inhibitor, was used to block the NF-κB signalling pathway. Cells were treated following the manufacturer’s instructions. The *cis*-reporter plasmids pISRE-Luc, pGAS-Luc, pAPl-Luc, and pNF-kB-Luc were purchased from Agilent Technologies Inc (Santa Clara, CA, USA). Antibodies used for analysis were anti-DEAD box protein 1 (DDX1; 11357-1-AP), anti-PABPC1 (10970-1-AP) (Proteintech, Rosemont, IL, USA), anti-IAV M1 antibody (GTX125928) (GeneTex, Irvine, CA, USA), and anti-IAV NS1 antibody (PA5-32243) (Invitrogen). Both RNAi MAX and Lipofectamine LTX were purchased from Invitrogen. For stimulation, the cells were incubated with BAY11-7082 or IFN-β for the indicated times following the manufacturer’s instructions. Transfection of A549 cells with poly(I: C) was performed using Lipofectamine LTX.

### Plasmids, small interfering RNAs, and cell transfection

The cDNA of LINC01197 and the CDS of PABPC1 were cloned into the pcDNA 3.1 vector, respectively. The small interfering RNA (siRNA)-specific sequence and siRNA universal negative control #1 (SIC001) was designed and synthesised by Sigma-Aldrich. Sequences of RNA oligos are 5′-GCUCUUAAGGGACAUGAAUdTdT-3′ (sense) and 5′-AUUCAUGUCCCUUAAGAGCdTdT-3′ (antisense) for LINC01197 and 5′-CAUGUAAGGUGGUUUGUGAdTdT-3′ (sense) and 5′-UCACAAACCACCUUACAUGdTdT-3’ (antisense) for PABPC1. For plasmid DNA transfection, cells were transiently transfected with the indicated plasmids in a six-well plate using Lipofectamine LTX according to the manufacturer’s instructions. Lipofectamine RNAiMAX reagent was used for siRNA transfection.

### RNA isolation, reverse transcription, and quantitative RT-PCR (qRT-PCR)

RNA was isolated using TRIzol reagent (Invitrogen) according to the manufacturer’s instructions and quantified using NanoDrop ND-1000 (Thermo Scientific, Waltham, MA, USA). The cDNA was synthesised using the PrimeScript 1st strand cDNA Synthesis kit (Takara, Dalian, China) with random or oligo (dT) primers. For quantitative mRNA and lncRNA expression analysis, qRT-PCR was performed using the SYBR premix Ex Taq II kit (Takara) according to the manufacturer’s instructions, and each assay was run in triplicate. The 2-ΔΔCt method was used to quantify the relative RNA levels against the housekeeping gene GAPDH. Primers for qPCR are listed in Additional file 1.

### Western blotting

For western blotting, the protein samples were denatured in an SDS-PAGE loading buffer at 100 °C for 10 min. Clarified lysates were resolved on 10% SDS-PAGE and transferred to 0.2 µM polyvinylidene fluoride (PVDF) membranes (Millipore, Billerica, MA, USA) using Turbo-Blot (Bio-Rad, Hercules, Ca, USA). Membranes were blocked with PBS supplemented with 5% (w/v) nonfat dry milk for 2 h and probed with primary antibodies overnight. Membranes were incubated with Chemiluminescent HRP substrate (Millipore), and protein signals were detected using Gel Doc XR + molecular imager (Bio-Rad).

### Luciferase assay

The LINC01197 promoter region was cloned into the pGL3-Basic luciferase vector (Promega, Madison, WI, USA). The indicated plasmids (firefly luciferase) and pRL-CMV plasmid (Renilla luciferase; Promega) were transfected into A549 cells, respectively. After transfection, cells were infected with IAV and harvested after 12 h to measure luciferase intensity. Luciferase reporter assay was performed using the Dual-Luciferase Reporter Assay System (Promega) according to the manufacturer’s instructions. Renilla luciferase activity was used to normalise the differences in transfection efficiency.

### Chromatin immunoprecipitation (ChIP)

ChIP analysis was performed using a commercially available ChIP assay kit (Millipore) according to the manufacturer’s instructions. Briefly, A549 cells were infected with PR8 for 12 h and were subjected to ChIP assays. The chromatin fraction was immunoprecipitated with 3 µg of anti-NF-κB P65 (Invitrogen; MA5-37156) or IgG control antibody (Millipore) overnight, and then chromatin complexes were captured with 20 µL magnetic beads at 4 °C for 4 h to overnight. After ChIP, purified DNA was cleaned using PCR purification columns (Qiagen). The genomic regions surrounding the transcription start site (TSS) of the indicated regions were amplified using primers for qPCR analysis. Additional file 2 lists the primer sequences used in ChIP investigations.

### Fluorescent in situ hybridization (FISH)

FISH assays were performed using FISH Tag Detection Kits (Invitrogen) in accordance with the manufacturer’s instructions. The template for transcribing anti-sense RNA and sense RNAs was generated by linearising the pEASY-T3 vector (TransGen Biotech, Beijing, China) expressing LINC01197.

### RNA pull-down assay and mass spectrometry

For RNA pull-down assay, a linearised pEASY-T3 vector expressing LINC01197 was used as a template to synthesise biotinylated LINC01197 or its antisense (AS) control RNA. Biotin-labelled sense or antisense RNAs were transcribed in vitro using the MAXIscript SP6/T7 kit (Invitrogen) and the Pierce™ RNA 3′ End Desthiobiotinylation kit (Thermo Scientific). The Pierce™ Magnetic RNA-Protein Pull-Down kit (Thermo Scientific) performed RNA pull-downs according to the manufacturer’s instructions. Protein eluates were separated by 10% SDS-PAGE and stained with ProteoSilver™ silver stain kit (Sigma-Aldrich). Trypsin digestion and band excision specific to LINC01197 were followed by LC–MS/MS analysis.

### RNA immunoprecipitation (RIP)

A549 cells were infected with PR8 at an MOI of 1 and collected for RIP at the indicated times. RIP was performed using the Magna RIP RNA-binding protein immunoprecipitation kit (Millipore) according to the manufacturer’s protocol. Briefly, cells were lysed in a lysis buffer containing RNase inhibitor and protease inhibitor. Protein A/G magnetic beads were incubated with antibodies against PABPC1 or IgG for 2 h at room temperature and further incubated with cell lysates by rotating at 4 ℃ overnight. After washing five times with lysis buffer, the co-precipitated RNA was extracted with Trizol (Invitrogen) for later qRT-PCR analysis.

### Co-immunoprecipitation (Co-IP)

HEK293T cells were transfected with the indicated plasmids. The cells were then infected with PR8 for 24 h and lysed with IP lysis buffer. To immunoprecipitate the protein complexes, the lysates were incubated with anti-PABPC1 antibody at 4 °C overnight. Meanwhile, control IgG was added to the lysate as a negative control. Protein A/G magnetic beads (Thermo Scientific) were added and rotated at 4 °C overnight. The immunoprecipitates were then blotted with the appropriate Ab against the protein as indicated.

### Statistical analysis

The results of the experiments were presented as means ± standard error of the mean (SEM). The means of the groups came from at least three independent experiments and were compared using the Student *t*-test (unpaired) or ANOVA, when appropriate. Differences were considered statistically significant with *p* < 0.05.

## Results

### LINC01197 is induced by IAV infection in a time- and dose-dependent manner

**Figure 1:**
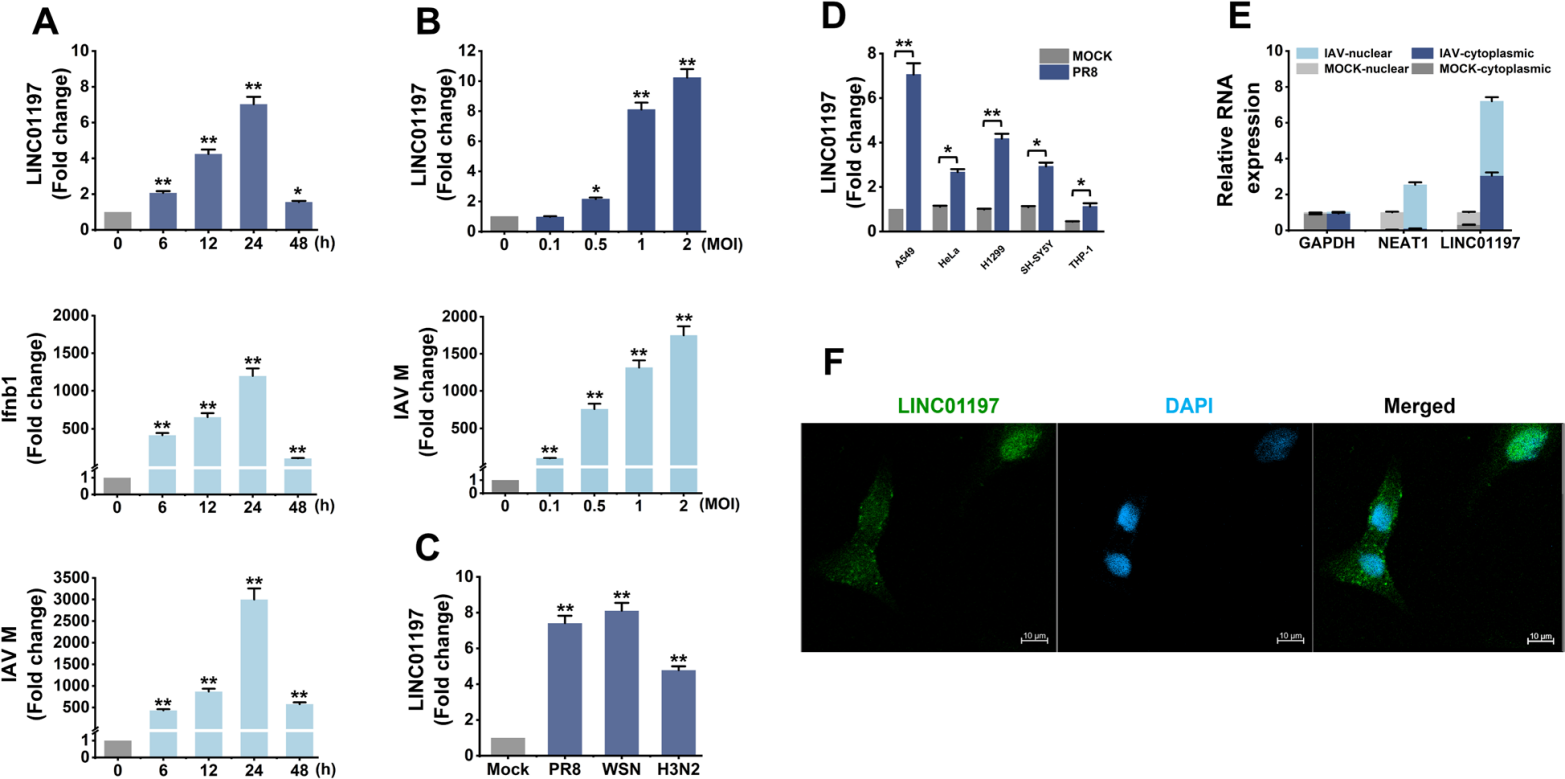
**LINC01197 is induced by IAV infection in a time- and dose-dependent manner.** **A** A549 cells were infected with IAV strain H1N1(PR8) for the indicated times. Total RNA was extracted and subjected to qRT-PCR to analyse the expression levels of LINC01197, Ifnb1, and IAV M. **B** A549 cells were infected with PR8 at different MOIs, including 0.1, 0.5, 1, and 2, for 24 h. Then, the relative levels of the LINC01197 and IAV M mRNA were detected by qPCR. **C** The LINC01197 expression in A549 cells infected with different IAV strains for 24 h was examined by qRT-PCR. **D** The LINC01197 expression in the indicated cell lines infected with PR8 for 24 h was determined by qRT-PCR. **E** A549 cells were infected with or without IAV for 24 h. After subcellular fractionation, the indicated RNA levels in cytoplasmic and nuclear extracts were measured by qRT-PCR. GAPDH mRNA and NEAT1 RNA were used as controls for cytoplasmic and nuclear RNA, respectively. Total RNA was used as an input control. **F** RNA FISH and confocal images showed the localisation of LINC01197 (in green) in A549 cells. Nuclei were stained with DAPI (blue). Scale bar: 10 μm. All qRT-PCRs shown are representative of three independent experiments with similar results. The results were normalised to GAPDH. Data are shown as means ± SEM. *p < 0.05; **p < 0.01 vs. 0 h, or mock-treated cells.

We first investigated whether IAV infection affects LINC01197 expression. To this end, we performed a time course analysis of LINC01197 expression on A549 cells infected with IAV. LINC01197 levels were determined by qRT-PCR. The results showed that LINC01197 expression was upregulated as early as 6 h post-infection (hpi), and its level continued to increase as the IAV infection progressed (Figure 1A, upper panel). Meanwhile, the upregulation of interferon beta 1 (Ifnb1) and IAV M mRNA expression was used to confirm active viral infection (Figure 1A, lower panels). Then, A549 cells were infected with IAV at different MOIs. LINC01197 was upregulated in an IAV dose-dependent manner, with a tenfold increase at the highest MOI infection (Figure 1B). In addition to the influenza A/PR/8/34 (H1N1) virus, infections with other IAV stains, including A/WSN/33(H1N1) and A/Lufang/9/93 (H3N2), also significantly stimulated the expression of LINC01197 in A549 cells (Figure 1C). To further investigate whether LINC01197 expression was induced by invading IAV in different cell lines, H1299, THP-1, SH-SY5Y, and Hela cells were used. Like the data obtained from A549 cells, LINC01197 was indeed elevated by IAV infection in H1299 cells, with a moderate increase in Hela, THP-1, and SH-SY5Y cells (Figure 1D).

Next, we examined its subcellular localisation by performing subcellular fractionation and analysing levels of LINC01197 by qRT-PCR. This analysis showed that LINC01197 was localised in both nuclear and cytosolic compartments of A549 cells. Following IAV infection, an increase of approximately 10% in the cytoplasmic portion of total LINC01197 was observed (Figure 1E). As expected, the GAPDH mRNA was found in the cytosol, whereas NEAT1 (a nuclear lincRNA) was specifically located in the nucleus. The localisation of LINC01197 was also confirmed by RNA FISH in A549 cells (Figure 1F). Together, these data demonstrate that IAV infection upregulates the expression of LINC01197.

### IAV-induced LINC01197 expression is associated with innate immune signalling

**Figure 2:**
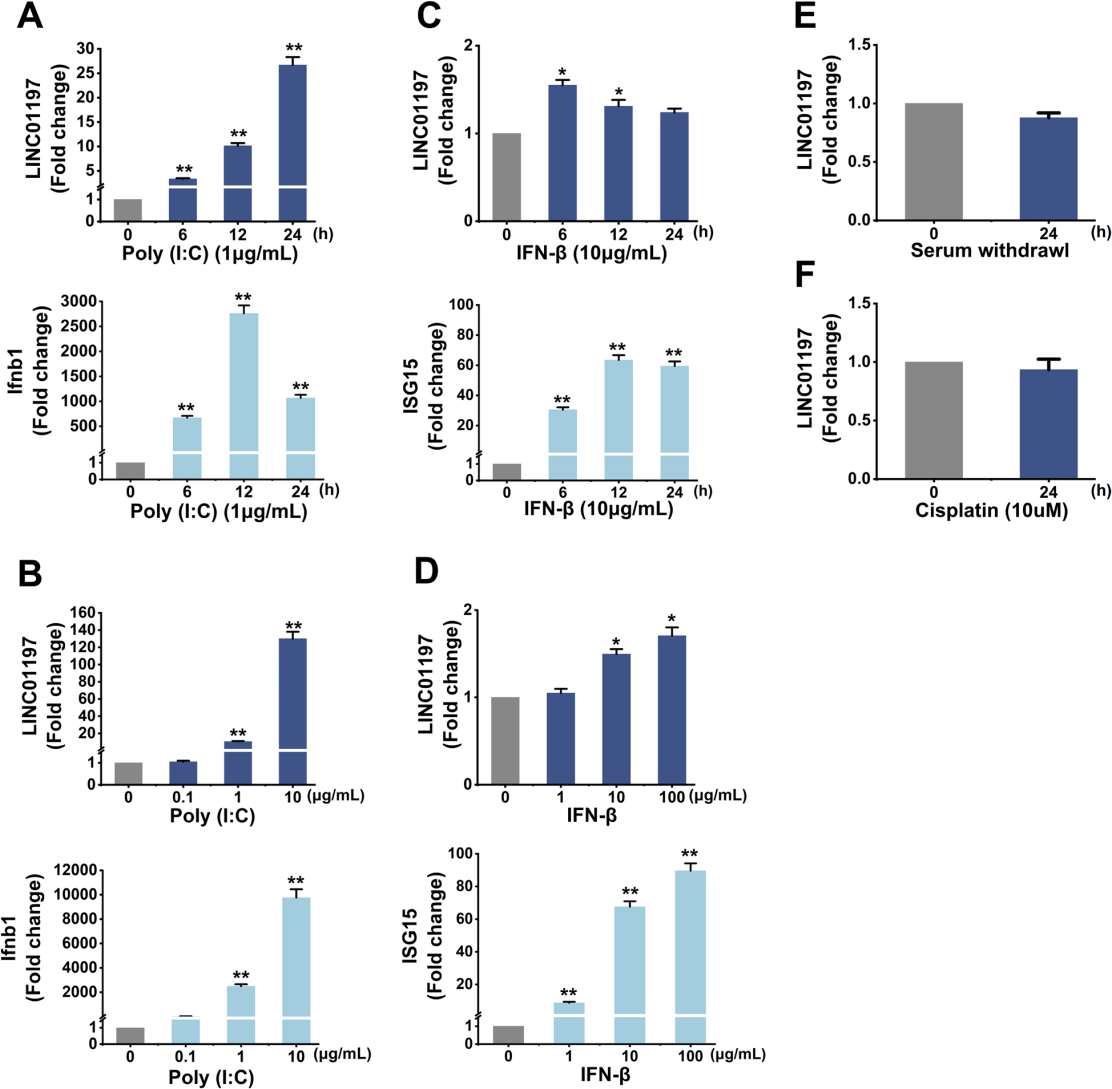
**IAV-induced LINC01197 expression is associated with innate immune signalling.** **A** The expression of LINC01197 and Ifnb1 in A549 cells transfected with poly(I: C) at a dose of 1 µg/mL was detected by qRT-PCR. **B** The levels of LINC01197 and Ifnb1 expression in A549 cells transfected with different concentrations of poly(I: C) for 12 h were determined by qRT-PCR. **C** A549 cells were stimulated with 10 µg/mL IFN-β and RNA was isolated for the times indicated. QRT-PCR measured the levels of LINC01197 and ISG15. **D** A549 cells were treated with different amounts of IFN-β for 6 h. QRT-PCR detected the levels of LINC01197 and ISG15. **E** The LINC01197 expression in A549 cells cultured in serum-free media for 24 h was examined by qRT-PCR. **F** A549 cells were treated with cisplatin for 24 h. QRT-PCR determined the expression of LINC01197. All shown qRT-PCR results represent three independent experiments with similar results. The results were normalised to GAPDH. Data are shown as means ± SEM. *p < 0.05; **p < 0.01 vs. 0 h, or control.

Since an active innate immune response accompanied upregulated LINC01197 expression, we speculated that innate immune signalling might be involved in precisely regulating LINC01197 transcription within infected host cells in response to IAV infection. To test this possibility, we transfected poly(I: C), a simulative stimulus of virus RNA, into A549 cells to stimulate cells. As expected, LINC01197 expression in poly(I: C)-stimulated A549 cells was dose- and time-dependent (Figures 2A, B). As a positive control, Ifnb1 mRNA levels dramatically increased in the A549 cells in response to poly(I: C) treatment. Of note, LINC01197 could be slightly upregulated by IFN-β (Figures 2C, D) and IFN-α (Additional files 3A, B) in a time- and dose-dependent manner. ISG15 mRNA served as a positive control. Serum supplies essential components such as growth factors, hormones, and amino acids to cell cultures, which can affect cellular phenotypic characteristics [17]. Next, we tested the effect of serum starvation on LINC01197 expression. The result showed that serum deprivation did not affect the LINC01197 transcription (Figure 2E). In addition, LINC01197 levels were not altered after stimulation with cisplatin (inducer of apoptosis) (Figure 2F). These data suggest that the induction of LINC01197 after exposure to IAV is associated with host innate immune responses independent of type I IFN signalling.

### IAV-induced expression of LINC01197 involves the NF-κB pathway

**Figure 3:**
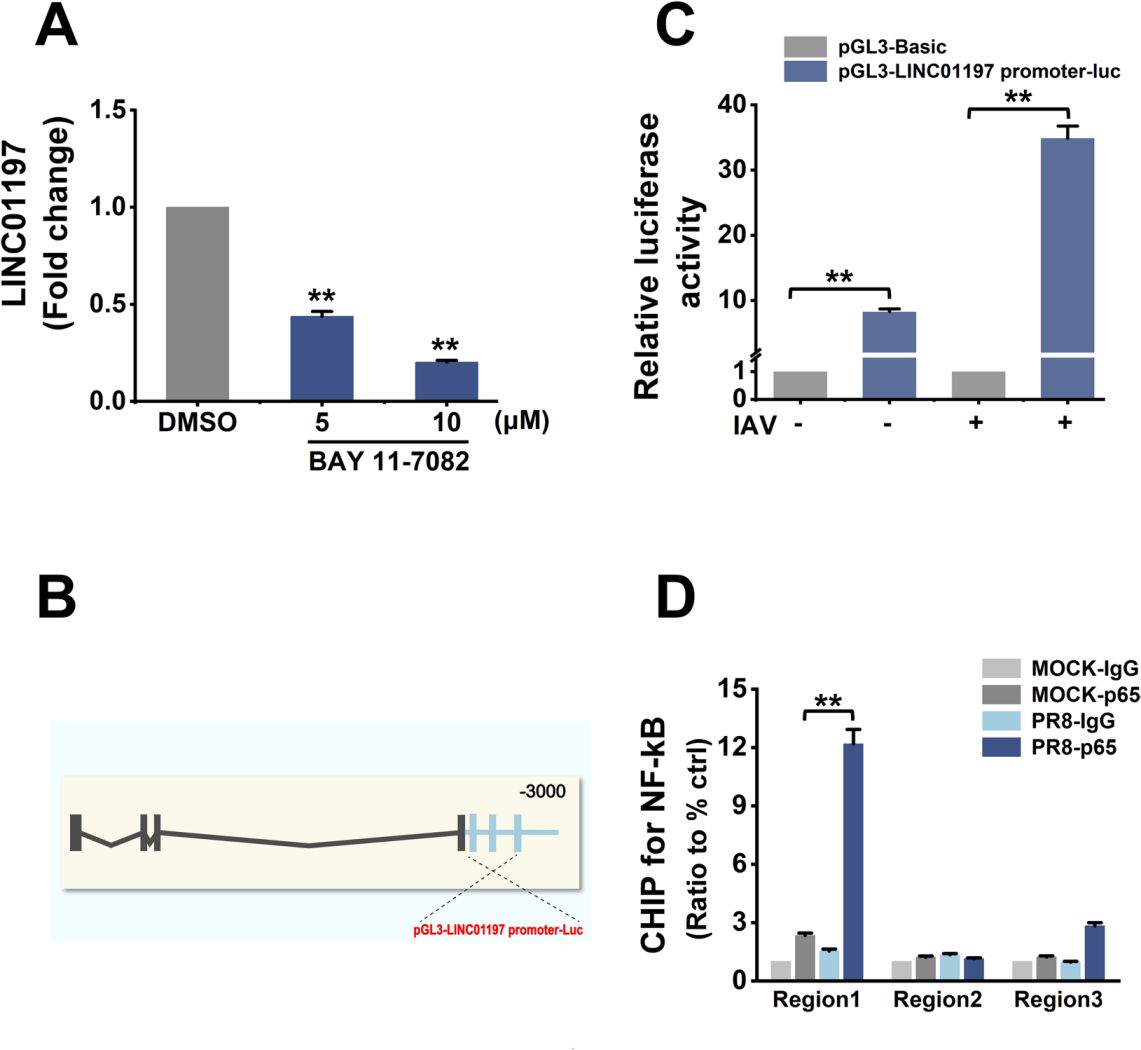
**IAV-induced expression of LINC01197 involves NF-κB pathway**. **A** A549 cells were incubated with different concentrations of BAY11-7082 or the DMSO control for 30 min before IAV infection. The expression levels of LINC01197 in A549 cells infected with IAV for 24 h were assessed by qRT-PCR. **B** Schematic representation of the predicted binding sites for NF-κB on 3 kb upstream of the transcription start site of LINC01197 for ChIP and luciferase assay. The blue box shows the potential binding site of NF-κB. **C** The LINC01197 promoter was cloned and inserted into the pGL3-Basic luciferase reporter construct. After co-transfection with the indicated plasmids and pRL-CMV, A549 cells were infected with/without IAV for 12 h. Cell lysates were harvested for the luciferase assay. The results were normalised to Renilla luciferase activity. Data are shown as means ± SEM. **p < 0.01 vs. pGL-Basic. **D** ChIP assays were performed in A549 cells infected with IAV for 12 h using anti-p65 or IgG antibodies. Enrichment of p65 at LINC01197 promoters is shown relative to input DNA (% input). Data represent means ± SEM of *n* = 3 biological replicates and represent at least two independent experiments. **p < 0.01 vs. IgG.

Next, BAY11-7082, a well-known inhibitor of NF-κB signalling cascade, was used to test the relationship between NF-κB signalling and LINC01197 expression. A549 cells were incubated with BAY11-7082 or DMSO (control) and then inoculated with 1 MOI of IAV for 24 h. As shown in Figure 3A, BAY11-7082 significantly blocked the IAV-induced LINC01197 transcription in A549 cells compared to the control. Putative NF-κB binding sites were predicted within the region of the 3-kb upstream region upstream of the transcription start site (TSS) of the LINC01197 gene by using the ALGGEN-PROMO and JASPER website (Figure 3B). Then, the potential promoter region was cloned upstream of the firefly luciferase coding region. As shown in Figure 3C, IAV infection enhanced the luciferase activity in cells transfected with the luciferase construct encompassing the promoter region of the LINC01197 gene but not in cells transfected with the empty vector (EV) control. The LINC01197 promoter fragments were then cloned upstream of the firefly luciferase coding region of the pGL3-basic reporter vector. Furthermore, ChIP assays were performed to confirm the direct interaction between RelA/p65 and the LINC01197 promoter. The results showed a significant enrichment of p65 in the putative promoter region-1 (Chr15:95328758–95328770) of the LINC01197 gene locus in A549 cells after IAV infection (Figure 3D). These results suggest that the NF-κB signalling pathway regulates IAV-induced LINC01197 expression.

### Altering the expression of LINC01197 has distinct effects on IAV replication

**Figure 4:**
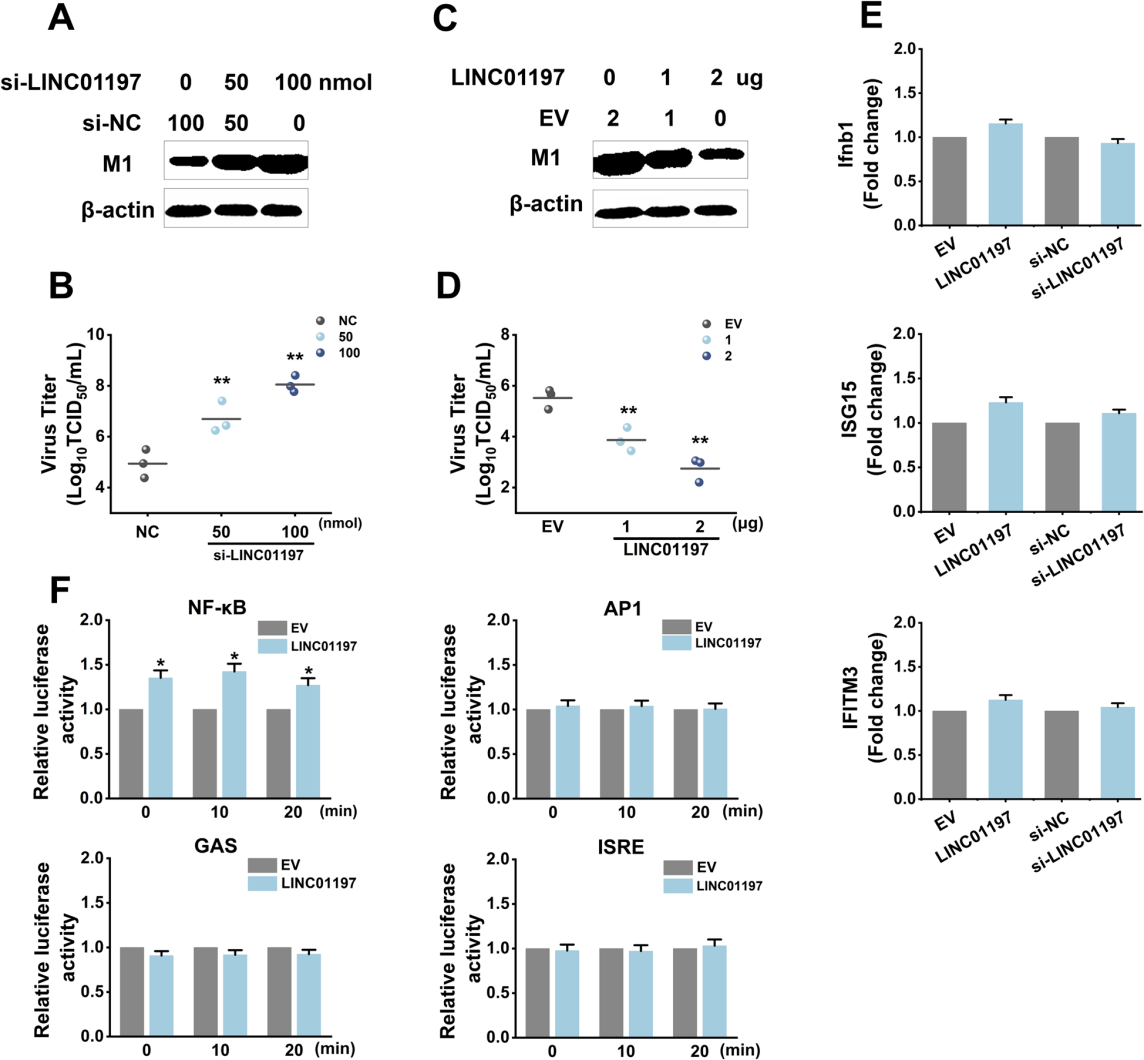
**Altering the expression of LINC01197 has distinct effects on IAV replication**. **A** A549 cells were transfected with increasing amounts of si-LINC01197 as indicated. NC-siRNA was added to bring the total siRNAs to 100 nmol. Cell lysates were collected at 36 hpi and subjected to western blot analysis to determine the levels of viral M1 proteins. **B** Culture supernatants were collected at 36 hpi. Viral productions were measured by a TCID_50_ assay performed on MDCK cells. **C** Increasing amounts of pcDNA3-LINC01197 were transfected into A549 cells. EV was added to raise the total plasmids to 2 µg. At 36 hpi, levels of viral M1 proteins were analysed by western blotting. **D** Virus titers in the supernatants were measured at 36 hpi. Data are shown as means ± SEM. **p < 0.01 vs. NC-siRNA or EV control. **E** A549 cells were transfected with the indicated plasmids or siRNAs. qRT-PCRs were performed to analyse the expression of Ifnb1, ISG15, and IFITM3. **F** The indicated plasmids and ISRE-, GAS-, AP1-, or NF-κB- reporter plasmids were co-transfected into A549 cells with pRL-CMV as a control. Twenty-four hours after transfection, the cells were infected with IAV. Dual luciferase assays were done at 12 hpi. Data are shown as means ± SEM. *p < 0.05 vs. EV.

The above-described data showed that IAV infection increased LINC01197 expression, and we wondered whether LINC01197 could reciprocally influence IAV replication. Compared with the cells transfected with negative control [NC] siRNA, A549 cells transfected with si-LINC01197 exhibited a significant increase in the production of IAV M1 protein, and M1 expression appeared to be related to the amount of si-LINC01197 applied (Figure 4A). Consistent with these results, through analysing the viral load of the 50% tissue culture infective dose (TCID_50_), we found that viral titres were increased after LINC01197 knockdown (Figure 4B). Next, we investigated whether overexpression of LINC01197 could suppress IAV replication. The results showed that the expression of IAV M1 protein and virus titers were limited in A549 cells in which LINC01197 was ectopically expressed in comparison to those transfected with the control vector in a dose-dependent manner (Figures 4C, D). Similar results were also obtained in H1299 cells (Additional file 4A).

Interestingly, the effects of LINC01197 on virus replication were not attributed to the host’s innate immune response, as the expression levels of Ifnb1 and effecter gene were not significantly altered by overexpression or knockdown of LINC01197 (Figure 4E). Meanwhile, the luciferase assay showed that LINC01197 overexpression did not significantly impact the luciferase activity of ISRE, AP1, and GAS. It also only exhibited a slight increase of NF-kB-p65-dependent luciferase activity after IAV infection (Figure 4F). In addition, we performed RNA-seq to profile the cellular transcriptional response to LINC01197 overexpression in A549 cells infected with PR8 for 24 h. Very few of the differentially expressed genes were found to be associated with pathogen infection, viral replication, or antiviral immune response through Gene Ontology (GO) enrichment analysis (data not shown), suggesting that LINC01197 probably restrains viral infection through a general mechanism. These data indicate that LINC01197 regulates IAV replication independent of the host antiviral innate immune responses.

### LINC01197 and PABPC1 physically interact following IAV infection but do not affect PABPC1 expression

**Figure 5:**
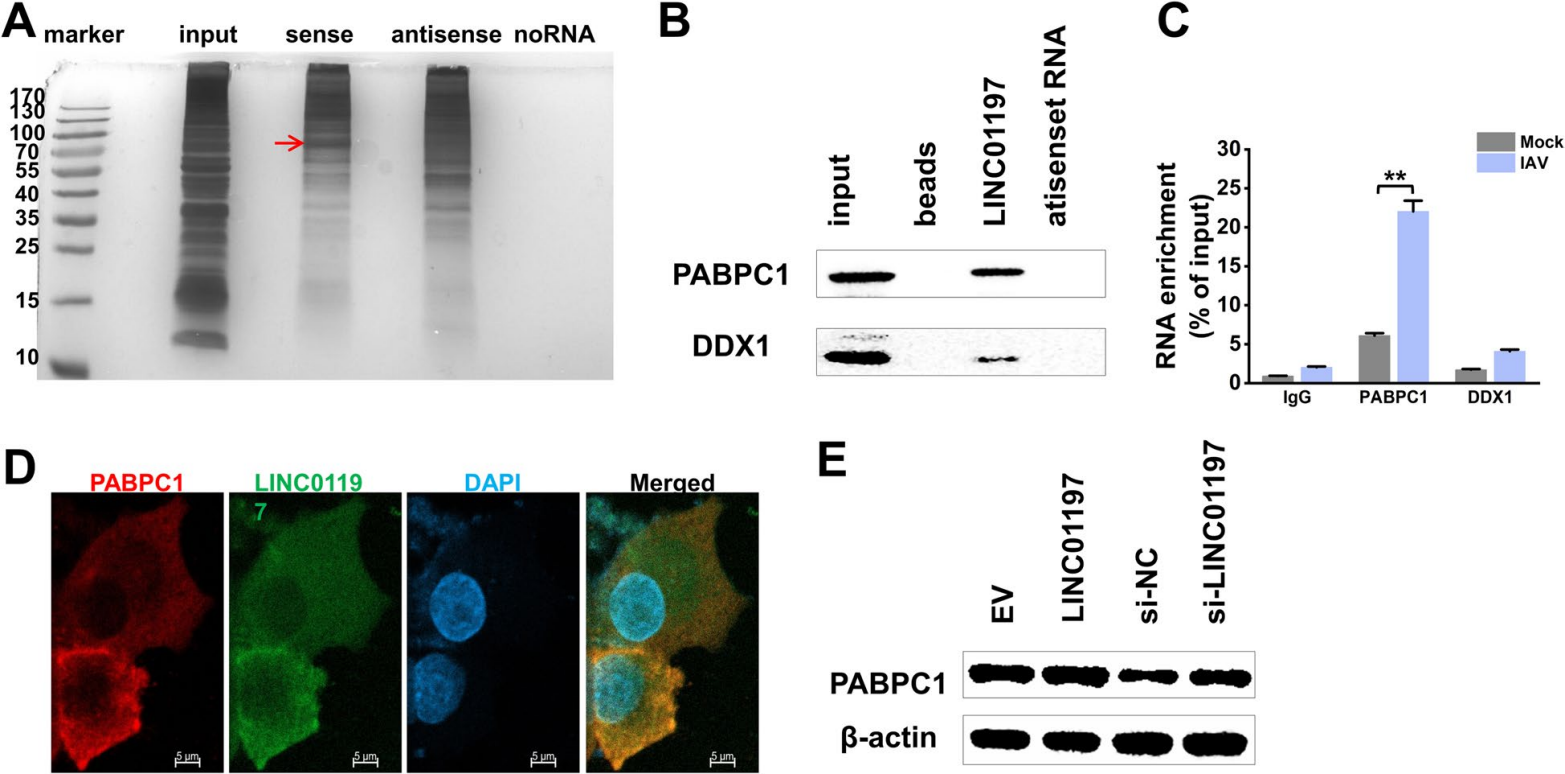
**LINC01197 physically interacts with PABPC1 following IAV infection but does not affect PABPC1 expression.** **A** Proteins purified from an in vitro binding assay using biotinylated LINC01197 or antisense control RNA and A549 extracts were separated by SDS-PAGE. Mass spectrometry further analysed the highlighted protein band. **B** RNA pull-down assay was performed to investigate the binding between LINC01197 and PABPC1 protein. The A549 cell lysates were incubated with in vitro transcribed LINC01197 or antisense control RNA. The pull-down complexes were then subjected to western blot analysis using the indicated antibodies. **C** RIP experiment was implemented to validate LINC01197 co-immunoprecipitated with PABPA1 protein. A549 cells were infected with IAV for 24 h. Cell lysates were immunoprecipitated with the indicated antibodies followed by qRT-PCR for LINC01197. IgG serves as the control for non-specific binding. Data are shown as means ± SEM.**p < 0.01 vs. mock-treated cells. **D** Confocal images showed the co-localisation of LINC01197 (in green) and PABPC1(in red) in A549 cells infected with IAV for 24 h. Nuclei were stained with DAPI (blue). Scale bar: 5 μm. **E** A549 cells were transfected with the indicated plasmids or siRNAs for 36 h, followed by western blot analysis for PABPC1.

To further understand the active mechanisms of LINC01197, we next sought to identify the protein partners of LINC01197. We first conducted RNA-protein binding assays by incubating in vitro transcribed biotinylated LINC01197 with cellular extracts from A549 cells. The antisense strand of LINC01197 served as the control RNA. We utilised streptavidin magnetic beads to capture the RNA-protein complexes and resolved them on SDS-PAGE. The protein bands, with a molecular weight of approximately 70 kDa, which exhibited significant enrichment in the LINC01197 pull-down, were further subjected to mass spectrometry for identification (Figure 5A). Using this approach, 39 RNA-binding proteins were identified in the LINC01197 pull-downs (Table 1). Western blot analysis was performed on the RNA pull-down samples to confirm the interaction of LINC01197 and PABPC1 (Figure 5B).

**Table 1:** **Top 20 of RNA-binding proteins pulled down by LINC01197**

Gene symbol	Description	Score	Matches	Sequences
PABPC1	Polyadenylate-binding protein 1 OS = Homo sapiens GN = PABPC1 PE = 1 SV = 2	319	21	16
DDX3X	ATP-dependent RNA helicase DDX3X OS = Homo sapiens GN = DDX3X PE = 1 SV = 3	309	21	14
HNRNPK	Heterogeneous nuclear ribonucleoprotein K OS = Homo sapiens GN = HNRNPK PE = 1 SV = 1	281	18	11
HSPA9	Stress-70 protein, mitochondrial OS = Homo sapiens GN = HSPA9 PE = 1 SV = 2	227	14	11
NCL	Nucleolin OS = Homo sapiens GN = NCL PE = 1 SV = 3	183	13	12
DDX17	Probable ATP-dependent RNA helicase DDX17 OS = Homo sapiens GN = DDX17 PE = 1 SV = 2	176	13	9
SYNCRIP	Heterogeneous nuclear ribonucleoprotein Q OS = Homo sapiens GN = SYNCRIP PE = 1 SV = 2	184	13	9
XRCC6	X-ray repair cross-complementing protein 6 OS = Homo sapiens GN = XRCC6 PE = 1 SV = 2	146	10	9
RPN1	Dolichyl-diphosphooligosaccharide–protein glycosyltransferase subunit 1 OS = Homo sapiens GN = RPN1 PE = 1 SV = 1	130	10	10
CKAP4	Cytoskeleton-associated protein 4 OS = Homo sapiens GN = CKAP4 PE = 1 SV = 2	120	9	9
IGF2BP1	Insulin-like growth factor 2 mRNA-binding protein 1 OS = Homo sapiens GN = IGF2BP1 PE = 1 SV = 2	126	9	8
EIF3L	Eukaryotic translation initiation factor 3 subunit L OS = Homo sapiens GN = EIF3L PE = 1 SV = 1	121	8	7
FARSB	Phenylalanine–tRNA ligase beta subunit OS = Homo sapiens GN = FARSB PE = 1 SV = 3	95	8	8
SRP68	Signal recognition particle subunit SRP68 OS = Homo sapiens GN = SRP68 PE = 1 SV = 2	85	6	6
HNRNPU	Heterogeneous nuclear ribonucleoprotein U OS = Homo sapiens GN = HNRNPU PE = 1 SV = 6	70	6	5
AHNAK	Neuroblast differentiation-associated protein AHNAK OS = Homo sapiens GN = AHNAK PE = 1 SV = 2	69	5	5
DDX21	Nucleolar RNA helicase 2 OS = Homo sapiens GN = DDX21 PE = 1 SV = 5	68	5	4
SRP72	Signal recognition particle subunit SRP72 OS = Homo sapiens GN = SRP72 PE = 1 SV = 3	59	4	4
EIF2AK2	Interferon-induced, double-stranded RNA-activated protein kinase OS = Homo sapiens GN = EIF2AK2 PE = 1 SV = 2	76	4	3
CPSF6	Cleavage and polyadenylation specificity factor subunit 6 OS = Homo sapiens GN = CPSF6 PE = 1 SV = 2	68	4	3

Since the RNA pull-down assay was performed in cell lysate rather than in a native cellular context, it is important to consider the potential artefact of the interaction between LINC01197 and PABPC1. To investigate this further, RIP assays were carried out by incubating PABPC1-specific antibodies with A549 lysates to isolate the endogenous protein, followed by qRT-PCR analysis to determine the levels of LINC01197 obtained from each immunoprecipitation. The results showed that LINC01197 was present in PABPC1 precipitate, further validating the interaction between LINC01197 and PABPC1 protein (Figure 5C). DDX1 was used as an RNA-binding protein control. FISH analysis provided additional evidence for the interaction between LINC01197 and PABPC1. A distinct co-localisation of LINC01197 and PABPC1 protein was observed in A549 cells following IAV infection (Figure 5D). Previous studies have shown that lncRNAs can interact with PABPC1, thereby influencing the expression of PABPC1 [18]. Given the interaction between LINC01197 and PABPC1, we investigated whether LINC01197 regulates PABPC1 expression. The results showed that PABPC1 protein levels were not altered by knockdown or overexpression of LINC01197 in A549 cells (Figure 5E). These results together demonstrate a specific interaction between LINC01197 and PABPC1.

### LINC01197 suppresses IAV replication via interaction with PABPC1

**Figure 6:**
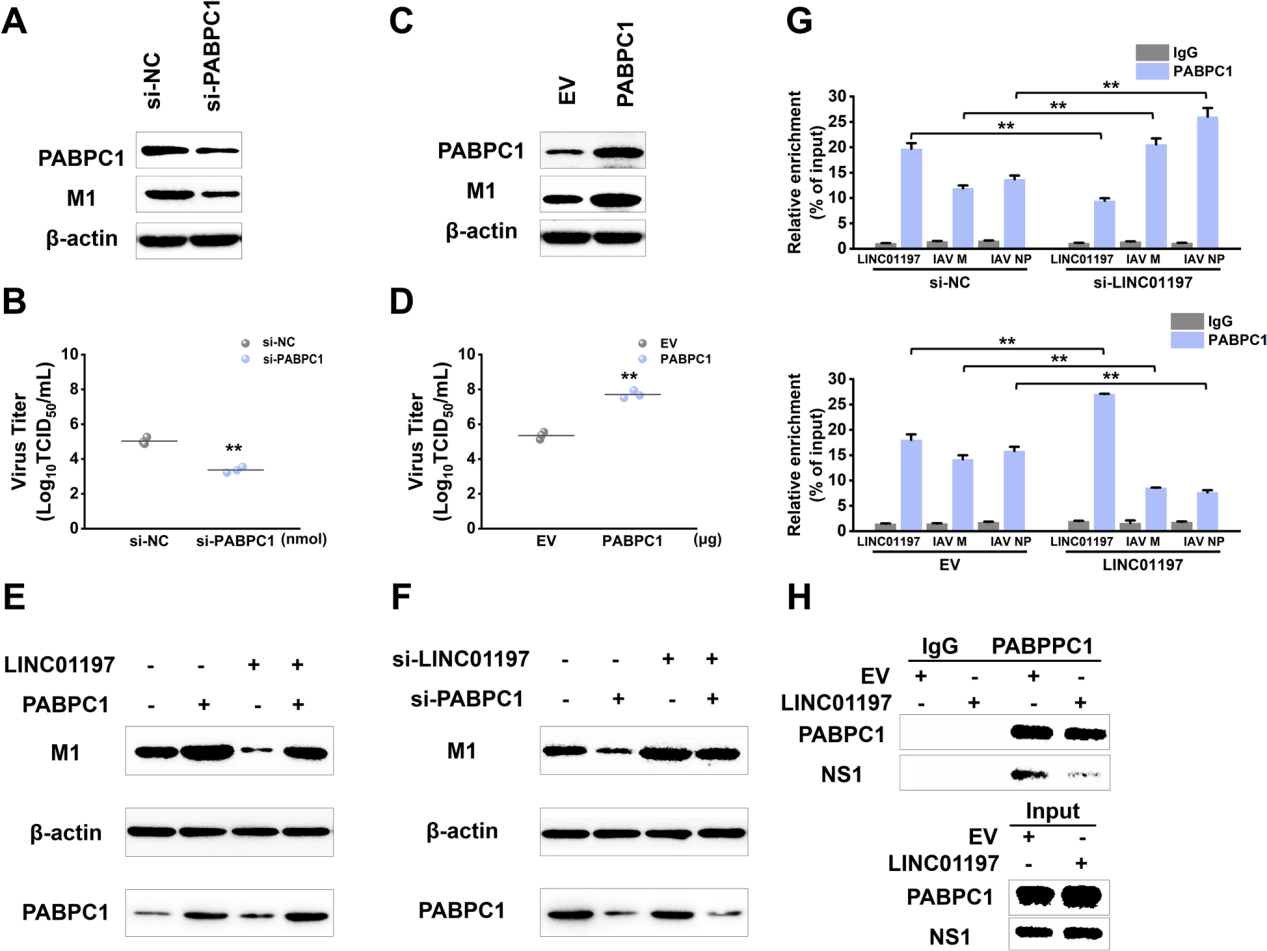
**LINC01197 suppresses IAV replication via interaction with PABPC1**. **A** A549 cells were transfected with si-PABPC1 or si-NC and then infected with IAV for 36 h. Cell lysates were obtained, and levels of PABPC1 and viral M1 proteins were detected using western blotting. **B** A TCID_50_ assay measured viral productions. Data represent mean ± SEM of *n* = 3 biological replicates. **p < 0.01 vs. si-NC. **C** A549 cells were transfected with pcDNA3-PABPC1 or pcDNA3 and then infected with IAV infection for 36 h. Cell lysates were subjected to western blot analysis to measure the levels of PABPC1 and viral M1 proteins. **D** Viral titres in the supernatants were determined by a TCID_50_ assay. Data represent mean ± SEM of *n* = 3 biological replicates. **p < 0.01 vs. EV. **E** A549 cells were transfected with the indicated siRNA for 24 h and then infected with IAV for 36 h. Levels of viral M1 and PABPC1 proteins in cell lysates were detected by western blotting. **F** A549 cells were transfected with the indicated plasmids for 24 h and then infected with IAV for 36 h. Levels of viral M1 and PABPC1 proteins in cell lysates were detected by western blotting. **G** A549 cells were transfected with the indicated plasmids or siRNAs for 24 h and then infected with IAV for 24 h. RIP assays were performed using a PABPC1-specific antibody followed by qRT-PCR for viral M1 and NP mRNAs. Data represent mean ± SEM of *n* = 3 biological replicates. **p < 0.01 vs. si-NC or EV. **H** A549 cells were transfected with the indicated plasmids and then infected with IAV for 24 h. Co-IP assays examined the interaction between PABPC1 and viral NS1 protein. Ten per cent of the input was loaded as a control.

Since previous studies have shown that PABPC1 drives the translation of IAV mRNA [19, 20], we tested whether the expression of PABPC1 affected IAV replication. The results showed a substantial decrease in IAV replication upon PABPC1 knockdown, as determined by western blotting and TCID_50_ assay (Figures 6A, B). Conversely, overexpression of PABPC1 increased infection load in A549 cells (Figures 6C, D). Comparable results were observed in H1299 cells following PABPC1 overexpression and knockdown (Additional file 4B). Next, we used a rescue experiment to confirm whether LINC01197 functions through PABPC1. As shown in Figure 6E, the supplementation of cells with recombinant PABPC1 attenuated the effect of LINC01197 overexpression on viral replication, as indicated by the M1 expression levels. Meanwhile, the impact of LINC01197 siRNA on IAV replication was partially alleviated in A549 cells when PABPC1 was silenced (Figure 6F). These data suggest that LINC01197 inhibits IAV replication, at least partially, by interacting with PABPC1.

**Figure 7:**
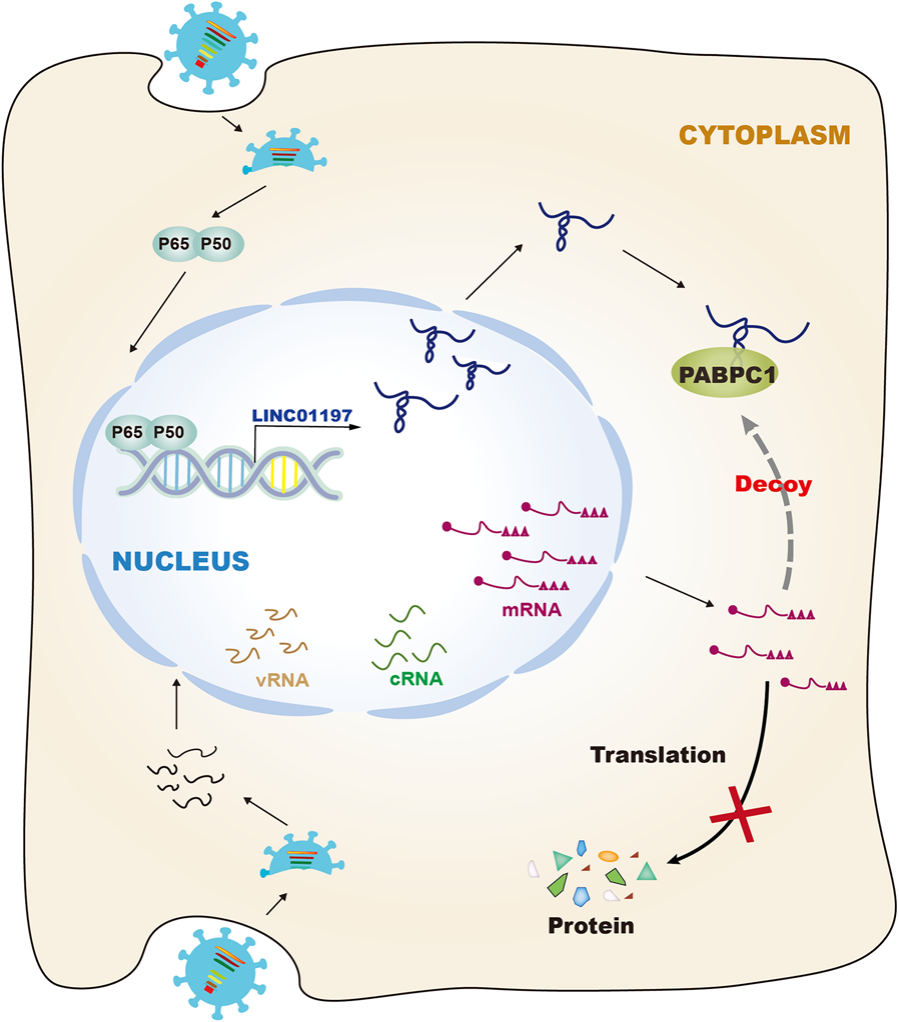
**Proposed molecular mechanism for the function of LINC01197 to attenuate IAV replication.** IAV infection significantly increases the expression of LINC01197, which is regulated by the NF-κB signalling pathway. LINC01197 can inhibit IAV replication, at least partially, by acting as a decoy to sequester PABPC1 away from viral mRNAs.

Given that PABPC1 directly binds to the 5ʹ UTRs of mRNAs from all eight segments of IAV [21], we next analysed whether the overexpression and knockdown of LINC01197 affected the interaction between PABPC1 and viral mRNAs as determined by the RIP assay. As shown in Figure 6G, the overexpression of LINC01197 resulted in a lower level of enrichment of M1 mRNA by PABPC1 IP. Conversely, silencing the endogenous expression of LINC01197 increased the binding of M1 mRNA to PABPC1. Furthermore, it has been previously demonstrated that the interactions of IAV non-structural protein 1 (NS1) and PABPC1 within the viral mRNAs promote the specific recruitment of the 43 S to viral mRNAs and allow the preferential translation of viral mRNAs [19]. Accordingly, we investigated the potential of LINC01197 to interfere with the PABPC1-NS1 interaction using a Co-IP experiment. The results showed that the transfection of pcDNA3-LINC01197 significantly disrupted the interaction between PABPC1 and viral NS1 (Figure 6H). Collectively, these data indicate that LINC01197 serves as a decoy to interact with PABPC1, thereby interfering with its function competitively (Figure 7).

## Discussion

LincRNAs are transcribed from the intergenic regions of annotated protein-coding genes without overlapping with protein-coding genes. Multiple lines of evidence have shown that LINC01197 is associated with diverse pathological and physiological processes. As early as 2017, LINC01197 was identified as a valuable diagnostic marker for lung squamous cell carcinoma [22]. Two years later, Ling et al. demonstrated that LINC01197 functions as a tumour suppressor and inhibits the proliferation of pancreatic ductal adenocarcinoma cells. Mechanistically, LINC01197 was found to act as a protein scaffold that promotes the assembly of a unique functional epigenetic complex involved in the regulation of gene expression [23]. Furthermore, LINC01197 can alleviate rheumatoid arthritis, a common systemic autoimmune disease, by sponging miR-150 to promote TLR4/NF-κB inactivation [24]. In 2021, Ducoli et al. renamed LINC01197 to LETR1 (lymphatic endothelial transcriptional regulator lncRNA 1). They revealed that LINC01197 modulates the expression of genes involved in the proliferation and migration of lymphatic endothelial cells through key epigenetic factors such as SEMA3C and KLF4 [25]. By binding to miR-516b-5p, LINC01197 inhibits CaOx-induced kidney stone formation [26]. Nevertheless, studies assessing the function of LINC01197 in viral infections are scarce.

Here, we present our findings that LINC01197, which is significantly induced by IAV infection, profoundly affects IAV replication. Furthermore, the results of a series of experiments demonstrate that LINC01197 interacts with PABPC1, a highly conserved multifunctional RNA-binding protein primarily associated with the regulation of mRNA translation and stability [27]. Several studies have demonstrated that PABPC1 plays a vital role in viral replication. For example, PABPC1 binds to the 5ʹ UTR of IAV mRNAs, which serves to recruit eIF4G and the subsequent initiation factors to promote the translation of viral mRNAs [21]. The binding of PABPC1 to the non-polyadenylated 3’ untranslated region of dengue virus (DENV) RNA facilitates translation enhancement [28]. Porcine epidemic diarrhoea virus (PEDV) N protein interacts with the protein translation initiation factor PABPC1 and eIF4F, which in turn promotes protein translation and viral replication [29]. Accordingly, we postulate that LINC01197 may serve as a sponge to sequester PABPC1, implying that LINC01197 may restrict PABPC1’s roles in IAV replication. Subsequently, LINC01197 was found to compete with viral mRNA for PABPC1 binding. Additionally, the overexpression of LINC01197 disturbs the interaction between PABPC1 and the viral NS1 protein. These findings support that LINC01197 functions through the competition model.

This decoy model is observed in multiple other lncRNAs. Neuronal enhancer RNAs (eRNAs) can interact with the negative elongation factor (NELF)-E, one of the NELF subunits, and facilitate the transient release of NELF for nascent transcripts during gene activation [30]. LncRNA MEG3-4 competes with the proinflammatory cytokine interleukin-1β (IL-1β) mRNA for binding to miR-138, resulting in elevated levels of IL-1β and enhanced inflammatory responses during bacterial infection [31]. Acting as a decoy, lnc-Lsm3b competes with viral RNA for binding with RIG-I, which contributes to stabilising inactive RIG-I and preventing the initiation of RIG-I downstream signalling [32]. Thus, the decoy mechanism is probably a common strategy that lncRNAs use to manipulate their targets.

Although we have proved that LINC01197 functions as a decoy to sequester PABPC1, this does not provide a complete picture of how LINC01197 suppresses IAV replication. First, LINC01197 could interact with other proteins or miRNAs associated with viral infection. Second, the nuclear fraction of LINC01197 may also participate in regulating IAV replication. Third, more viruses need to be tested to determine the potential of LINC01197 as an antiviral host factor. These deserve further investigation in the future.

In conclusion, this is the first study to describe the role of LINC01197 in viral infection. IAV infection induces LINC01197 expression through the NF-κB pathway. Furthermore, LINC01197 acts as a decoy to sequester PABPC1 protein, attenuating viral replication. These findings provide new insights into the role of lncRNAs in host-virus interactions.

## Supplementary Information

**Additional file 1. Primers were used for quantitative real-time PCR and other assays in this study.**

**Additional file 2. Primers used for ChIP-qPCR.**

**Additional file 3. The expression of LINC01197 and ISG15 in response to IFN-α stimulation.** (A) After 10 ng/mL IFN-α stimulation of A549 cells, RNA was isolated at the indicated times. QRT-PCR determined the levels of ISG15 and LINC01197. (B) qRT-PCR was used to determine the expression levels of LINC01197 and ISG15 in A549 cells treated with different concentrations of IFN-α for 6 h.

**Additional file 4. Levels of IAV M1 protein in H1299 cells with the overexpression and knockdown of LINC01197 or PABPC1.** H1299 cells were transfected with the indicated plasmids or siRNAs for 24 h to achieve the overexpression and knockdown of LINC01197 (A) or PABPC1 protein (B). Following IAV infection for 36 h, cell lysates were harvested, and the levels of viral M1 proteins were analysed using western blotting.

## Data Availability

All data generated or analysed during this study are included in this published article and its supplemental information files.
