# Supplementary material for: LINC01197 inhibits influenza A virus replication by serving as a PABPC1 decoy
Source: Vet Res. 2024 Sep 27;55:121. doi: 10.1186/s13567-024-01379-7 (PMC11430458; doi:10.1186/s13567-024-01379-7)
Supplement: Supplementary file 1 — Additional file 1. Primers were used for quantitative real-time PCR and other assays in this study. [file 13567_2024_1379_MOESM1_ESM.docx]

**Additional file 1 Primers used for quantitative real time-PCR and other assays in this study.**

| Gene Symbol | Forward primer (5’→3’) | Reverse primer (5’→3’) |
| --- | --- | --- |
| Ifnb1 | AGTAGGCGACACTGTTCGTG | GCCTCCCATTCAATTGCCAC |
| GAPDH | CAATGACCCCTTCATTGACC | TTGATTTTGGAGGGATCTCG |
| β-Actin | ACCTTCTACAATGAGCTGCG | CCTGGATAGCAACGTACATGG |
| IAV-M | GACCAATCCTGTCACCTCTGA | GTATATGAGGCCCATRCAACTG |
| ISG15 | CAGCCATGGGCTGGGAC | CTTCAGCTCTGACACCGACA |
| IFITM3 | CATCGTCATCCCAGTGCTGAT | ATGGAAGTTGGAGTACGTGGG |
| LINC01197 | GCACCAATAGCAACCAAATCCA | ACAGCTTGGATACCGCTTCTT |
| NEAT1 | GTGGCTGTTGGAGTCGGTAT | TAACAAACCACGGTCCATGA |
