# Supplementary material for: LINC01197 inhibits influenza A virus replication by serving as a PABPC1 decoy
Source: Vet Res. 2024 Sep 27;55:121. doi: 10.1186/s13567-024-01379-7 (PMC11430458; doi:10.1186/s13567-024-01379-7)
Supplement: Supplementary file 2 — Additional file 2. Primers used for ChIP-qPCR. [file 13567_2024_1379_MOESM2_ESM.docx]

**Additional file 2. Primers used for ChIP-qPCR.**

| Region | Forward primer (5’→3’) | Reverse primer (5’→3’) |
| --- | --- | --- |
| 1 | CCACCTGCTACATTACCAAATAC | GTATTGTACCGCCCCATCCT |
| 2 | GGTAGTGGACATGGCAGAGG | AGTCCTGCAGGTGCTGATTC |
| 3 | AGTCCGAAATTATTCGGGGGTT | CAAAAGGATGGGGCTTGGAATG |
