# Supplementary figures and images for: LINC01197 inhibits influenza A virus replication by serving as a PABPC1 decoy
Source: Vet Res. 2024 Sep 27;55:121. doi: 10.1186/s13567-024-01379-7 (PMC11430458; doi:10.1186/s13567-024-01379-7)

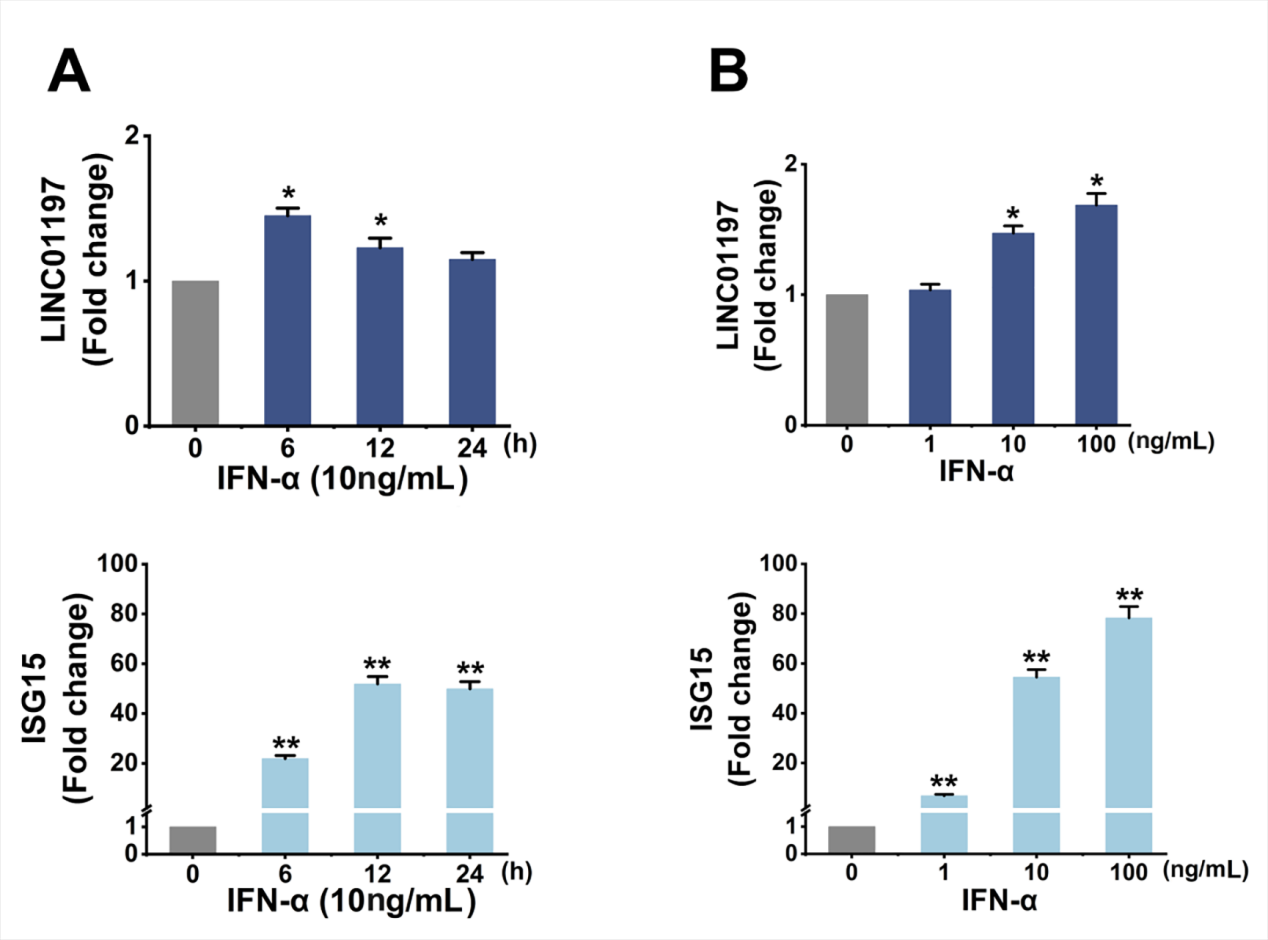

Supplement: Supplementary file 3 — Additional file 3. The expression of LINC01197 and ISG15 in response to IFN-α stimulation. (A) After 10 ng/mL IFN-α stimulation of A549 cells, RNA was isolated at the indicated times. QRT-PCR determined the levels of ISG15 and LINC01197. (B) qRT-PCR was used to determine the expression levels of LINC01197 and ISG15 in A549 cells treated with different concentrations of IFN-α for 6 h. [file 13567_2024_1379_MOESM3_ESM.docx]

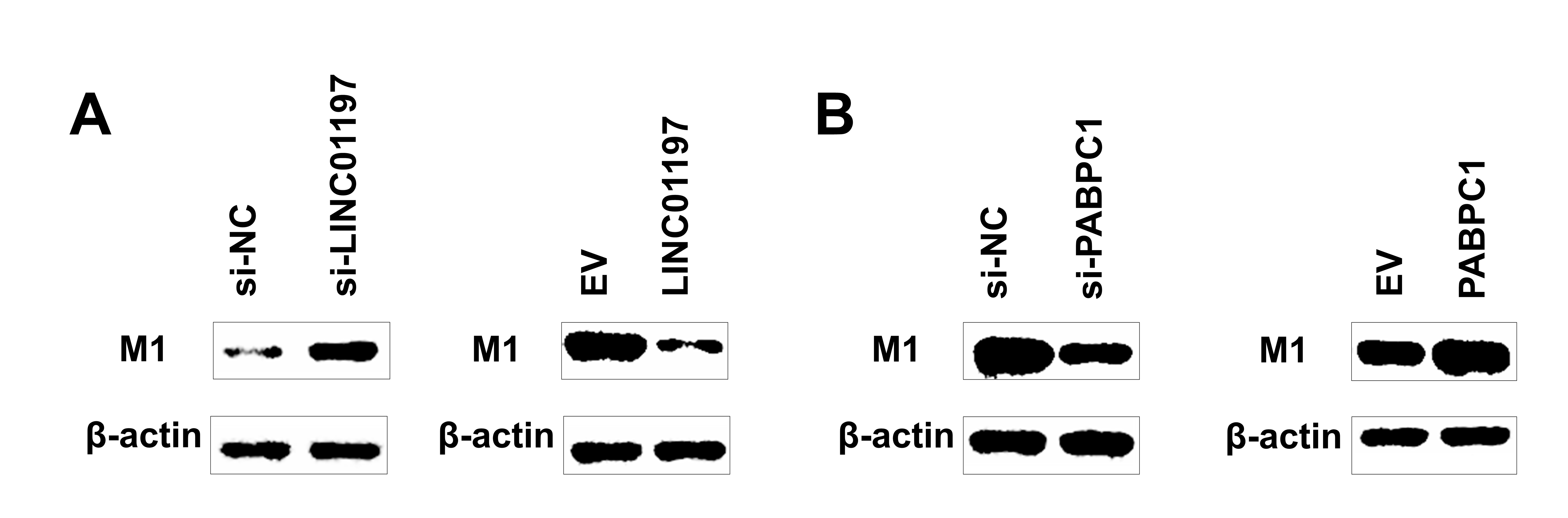

Supplement: Supplementary file 4 — Additional file 4. Levels of IAV M1 protein in H1299 cells with the overexpression and knockdown of LINC01197 or PABPC1. H1299 cells were transfected with the indicated plasmids or siRNAs for 24 h to achieve the overexpression and knockdown of LINC01197 (A) or PABPC1 protein (B). Following IAV infection for 36 h, cell lysates were harvested, and the levels of viral M1 proteins were analysed using western blotting. [file 13567_2024_1379_MOESM4_ESM.docx]
